# Seasonal Effect on Bacterial Communities Associated with the Rhizospheres of *Polhillia*, *Wiborgia* and *Wiborgiella* Species in the Cape Fynbos, South Africa

**DOI:** 10.3390/microorganisms10101992

**Published:** 2022-10-09

**Authors:** Tiisetso Mpai, Sanjay K. Jaiswal, Christopher N. Cupido, Felix D. Dakora

**Affiliations:** 1 Department of Crop Sciences, Tshwane University of Technology, Pretoria 0001, South Africa; 2 Department of Chemistry, Tshwane University of Technology, Arcadia Campus, Private Bag X680, Pretoria 0001, South Africa; 3 Department of Botany, University of Fort Hare, Campus Ring Road, Alice 5700, South Africa

**Keywords:** microbiome, 16S rRNA, phylum, genera, shrub legumes, bacteria

## Abstract

The Cape fynbos biome in South Africa is home to highly diverse and endemic shrub legumes, which include species of *Aspalathus*, *Polhillia*, *Wiborgia* and *Wiborgiella*. These species play a significant role in improving soil fertility due to their ability to fix N_2_. However, information regarding their microbiome is still unknown. Using the 16S rRNA Miseq illumina sequencing, this study assessed the bacterial community structure associated with the rhizospheres of *Polhillia pallens*, *Polhillia brevicalyx*, *Wiborgia obcordata*, *Wiborgia sericea* and *Wiborgiella sessilifolia* growing at different locations during the wet and dry seasons in the Cape fynbos. The results showed that the most dominant bacterial phylum was Actinobacteria during both the dry (56.2–37.2%) and wet (46.3–33.3%) seasons. Unclassified bacterial genera (19.9–27.7%) were the largest inhabitants in the rhizospheres of all five species during the two seasons. The other dominant phyla included Bacteroidetes, Acidobacteria, Proteobacteria and Firmicutes. *Mycobacterium* and *Conexibacter* genera were the biggest populations found in the rhizosphere soil of all five test species during both seasons, except for *W. obcordata* soil sampled during the dry season, which had *Dehalogenimonas* as the major inhabitant (6.08%). In this study plant species and growth season were the major drivers of microbial community structure, with *W. obcordata* having the greatest influence on its microbiome than the other test species. The wet season promoted greater microbial diversity than the dry season.

## 1. Introduction

South Africa is a biologically diverse country with nine terrestrial biomes. The fynbos biome is located at the southwestern tip of Africa, and recognized as one of the world’s biological hotspots due to its high plant diversity and endemicity, including shrub legumes [1,2,3,4]. The high diversity of nodulated legume species in the Cape fynbos region may suggest that this region is a hotspot for bacterial diversity [5,6,7]. Shrub legumes such as *Polhillia*, *Wiborgia* and *Wiborgiella* grow naturally in the fynbos [8,9], and they are characterized by bright yellow and/ or white flowers, a major attraction for tourists. These plants can also be used as cut-flowers in the ornamentals industry. Furthermore, they have the potential for reforestation and for control of soil erosion, which may be caused by heavy rains and wind [10].

Similar to other shrub legumes in the Cape fynbos [5,11,12]; *Polhillia*, *Wiborgia* and *Wiborgiella* species are able to nodulate with a diversity of soil bacteria, including *Mesorhizobium* and *Rhizobium* species [13], indicating their potential in fynbos biodiversity improvement. Furthermore, they are reported to derive over 61% of their N nutrition from biological nitrogen fixation [8], which means they depend on soil microbes for survival. Moreover, these species are also known to exhibit high acid and alkaline phosphatase activity, which led to greater P availability in the rhizosphere and its increased uptake and accumulation in plant shoots [8]. However, the soils supporting the growth of these legumes are generally sandy and acidic, with a pH of 2 to 5 [9,14,15]. As a result, the fynbos soil is nutrient-poor, with low total N and available P [16,17].

Soil microbes, including bacteria, exist in rhizosphere soils, and thus their activity and diversity are most dominant in the rhizosphere than in the non-rhizosphere bulk soils [18,19]. Both wet and dry seasons are known to alter ecosystem functioning through changes in rhizosphere microbial community structure and diversity [20,21]. The interaction of these endemic legumes with microbes in the rhizosphere can influence soil biogeochemical processes, including organic matter turnover, production of indole acetic acid (IAA), as well as mineralization of nitrogen and phosphorus [22,23]. These processes probably help to suppress soil-borne pathogens, thus improving soil fertility and promoting species diversity [24]. 

Studies of microbial community structure associated with endemic plant species in the Cape fynbos [6,25,26,27,28] have revealed high microbial diversity belowground, similar to aboveground plant species diversity. Microbial diversity can however be altered by many factors, including soil pH, plant species/type, plant age, cultivation history, as well as growth season [6,25,26,27,28]. The rhizosphere of legumes in the fynbos can therefore differ from plant to plant and species to species during different seasons.

The aim of this study was to assess the diversity of bacterial communities in the rhizosphere soils of *Polhillia brevicalyx*, *Polhillia pallens*, *Wiborgia sericea*, *Wiborgia obcordata* and *Wiborgiella sessilifolia* growing at different locations in the Cape fynbos during both wet and dry seasons.

## 2. Materials and Methods

### 2.1. Site Description and Soil Sampling

Sampling of rhizosphere soils from *Polhillia, Wiborgia* and *Wiborgiella* species was performed during the dry (February–March, 2017) and wet (August–September, 2017) seasons at five different locations in the Cape fynbos, as indicated in Table 1. Three replicate soil samples were collected from 20 cm depth at each location, pooled, and thoroughly mixed to obtain a single representative soil sample per location. For each species, there were five soil samples per season. As shown in Table 2, 100 g soil was weighed from each sample and determine water content. Soil water content was determined using the gravimetric water content (*θ*_m_) [29]. Soil analysis was performed at the Institute for Plant Production, Elsenburg, in the Western Cape, for only the wet season (Table 3).

### 2.2. Total Bacterial Genomic DNA Extraction from Rhizosphere Soils

Total bacterial genomic DNA was extracted from each rhizosphere soil sample, as described by Jaiswal et al. [22]. Bacterial DNA was extracted from 0.5 g of each rhizosphere soil using PowerSoil™ DNA Isolation Kit (MO BIO Laboratories, Inc., Carlsbad, CA, USA), according to the manufacturer’s instructions. After determining the DNA concentrations using the Qubit™ 4 Fluorometer (Invitrogen, cat. #Q33226).

### 2.3. Library Preparation

Random fragments of the DNA samples were used to prepare the library, followed by 5′ and 3′ adapter ligation. PCR amplification was performed using adapter-ligated primer pairs 5′TCG TCG GCA GCG TCA GAT GTG TAT AAG AGA CAG CCT ACGGGNGGC WGC AG3′ and 5′GTC TCG TGG GCT CGG AGA TGT GTA TAA GAG ACA GGA CTACHVGGG TAT CTA ATC C3′ targeting variable regions V3-V4 of the 16S rRNA gene [30] (Klindworth et al. 2013) in 25 μL reaction volume containing 12.5 μL KAPA HiFi hotstart ready mix (2×), 5 μL each of forward and reverse primer (1 μM) and 2.5 μL sterilized double distilled water with the standard temperature profiles (30″ − 95 °C, 25 × (30″ − 95 °C, 30″ − 55 °C, 30″ − 72 °C), 5′ − 72 °C). The PCR-amplified products were purified using AMPure XP beads. The library was loaded onto a flow cell for cluster generation.

### 2.4. Sequencing and Data Assembling

Illumina SBS technology was employed for paired-end sequencing. During data analysis and alignment, the newly identified sequence reads were aligned using FLASH to the reference genome. The taxonomical abundance (%) of microbial communities in each rhizosphere soil was calculated using read files as queries against removed and de-replicated sets of sequences from the small subunit (SSU) UCLUST. After normalization of sequences of each sample, rarefaction analysis was performed at the species level for sampling adequacy using alpha_rarefaction.py of QIIME. The data were submitted to NCBI Sequence Read Archive SUB11923586 under Bioproject ID PRJNA880697.

## 3. Results

### 3.1. Site Elevation and Physical Properties of Rhizosphere Soils

**Figure 1:**
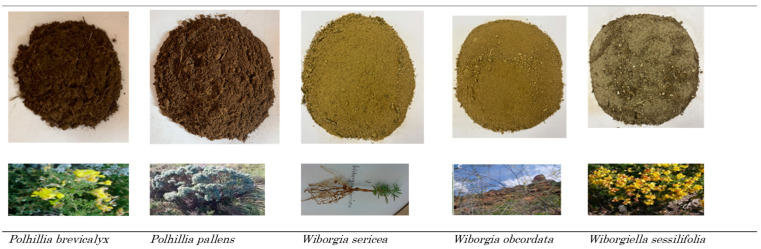
Physical appearance of the soil samples collected from different locations of Cape fynbos.

Uitvlucht and Ghwarriekop farms, where rhizosphere soils of *Polhillia brevicalyx* and *Polhillia pallens* were respectively collected, had an elevation of 310 and 152 m in that order. The soil texture was clayey at both locations (Figure 1). The water content from the rhizosphere of *P. pallens* and *P. brevicalyx* was, respectively, 9.26 and 8.21 % for the dry season and 26.55% and 25.47% for the wet season (Table 2). Soils from the rhizosphere of *Wiborgia sericea* and *Wiborgia obcordata* collected at Traveller’s Rest farm and Bushmans Kloof areas were sandy and at 120 and 441 m elevations, respectively. The gravimetric water contents were 6.37% and 20.0% for *W. sericea* in the dry and wet seasons, respectively, and 3.93% and 11.90% for *W. obcordata* in the dry and wet seasons, respectively (Table 2). The Bredasdorp area had loamy soil and was at 311 m elevation (Table 1). *Wiborgiella sessilifolia* rhizosphere soil recorded a water content of 5.42% (Table 2).

### 3.2. Chemical Properties of Rhizosphere Soils

Except for *Wiborgiella sessilifolia* rhizosphere soil from Bredasdorp, which had a pH of 8.2, the pH of all the other rhizosphere soils was acidic and ranged from pH 4.3 for *W. obcordata* to pH 5.5 for *P. brevicalyx*. The concentrations of macronutrients (namely, Mg, K, Na, P and C) were consistently higher in the rhizosphere soil of *P. pallens*, followed by *P. brevicalyx*, and much lower for *Wiborgia obcordata*. The concentration of soil micronutrients (Cu, Zn and B) mirrored those of the macronutrients, with high levels recorded for the rhizosphere soil of *P. pallens* and *P. brevicalyx* and lowest for *W. sericea* and *W. obcordata*. Fe concentration was generally high at all sites, with *P. pallens* showing the highest level (174.6 mg/kg), followed by *Wiborgia sericea* (129.2 mg/kg), with *Wiborgiella sessilifolia* the lowest (46.9 mg/kg). *Wiborgiella sessilifolia* recorded the highest NH_4_^+^ concentration (0.67 mg/kg), and *Wiborgia sericea* the lowest (0.03 mg/kg) (Table 2).

### 3.3. Quantity and Quality of Raw Sequences Generated

The quality and quantity of total sequences generated from the analysis were generally adequate. The total sequence bases and read counts were relatively higher for the rhizosphere soils collected during the wet season than the dry season. The least sequence bases recorded after quality control of all samples were from rhizosphere soils of *P. brevicalyx* (4,444,959 bp), followed by rhizosphere soils of *Wiborgiella sesilifollia* (4,521,971 bp); while the highest sequence bases were observed in the rhizosphere soils of *P. pallens* (5,537,882 bp) followed by *Wiborgia sericea* (5,180,741 bp). Furthermore, the reads outputs varied across samples, with ˃10,000 reads being obtained for each sample and the trend mirroring that of total sequence bases (Table S1). For example, the lowest read count was recorded for *P. brevicalyx* rhizosphere soil (11,820 bp), followed by *Wiborgiella sessilifolia* (12,006 bp), while the highest reads recorded were from *P. pallens* rhizosphere soils (14,904 bp). Moreover, Over 90 and 80% of the raw sequence bases passed the quality score of over 20 and 30%, respectively (Table S1)

### 3.4. Rhizosphere Bacterial Communities

#### 3.4.1. Polhillia Brevicalyx

**Figure 2:**
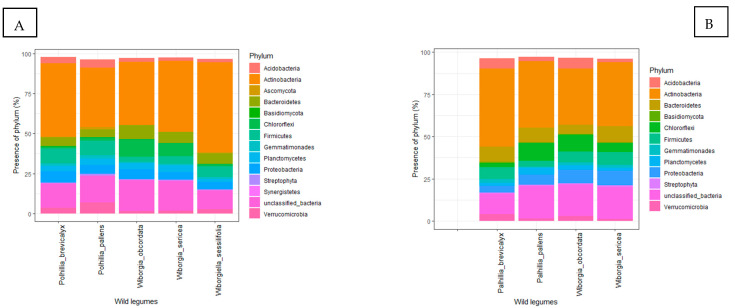
Taxonomic distribution based on 16S rRNA sequences described at phylum level in rhizosphere soils sampled during the (**A**) dry and (**B**) wet seasons.

Actinobacteria formed the largest population in the rhizosphere of *P. brevicalyx* and ranged from 45.8% to 46.3% in soil collected during the dry and wet seasons, respectively, followed by unclassified bacteria, which were 15.2% and 12.2% in the dry and wet seasons, respectively. During the dry season, Firmicutes (9.5%), Proteobacteria (7.0%) and Bacteroidetes (5.2%) showed relatively larger populations, in contrast to Bacteroidetes (9.1%), Firmicutes (6.9%) and Acidobacteria (6.0%) which recorded much larger populations during the wet season. Although they differed in population sizes, most of the bacteria found in the wet season were also available in the dry season (Figure 2).

**Figure 3:**
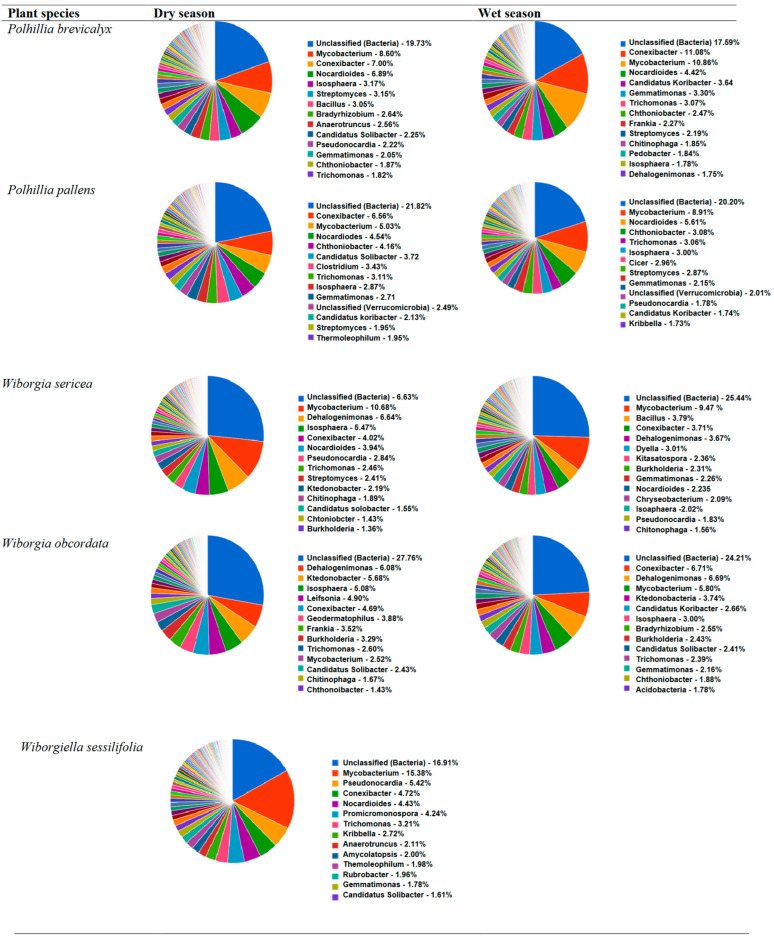
Taxonomic distribution based on 16S rRNA sequences described up to genus level in rhizosphere soils of *Polhillia brevicalyx* and *Polhillia pallens* sampled during the wet and dry seasons at the Cape fynbos biome, South Africa.

At the genera level, unclassified bacteria were dominant and formed about 19.7 and 17.5 %, respectively, in soils collected during the dry and wet seasons. *Mycobacterium*, *Conexibacter* and *Nocardioides*, respectively, recorded 8.6%, 7.0% and 6.8% in the dry season, and 10.8%, 11.0% and 4.4% in the wet seasons. Interestingly, *Bacillus*, *Bradyrhizobium*, *Anaerotruncus* and *Pseudonocardia* were only present in soils sampled during the dry season, while *Frankia*, *Chitinophaga*, *Pedobacter* and *Dehalogenimonas* were present in only soil collected during the wet season. Of the 13 genera identified, nine were found in both dry and wet seasons (Figure 3). From the rarefaction analysis, there was high species diversity in both seasons, as 2000 species could be counted using 140,000 sequence reads.

#### 3.4.2. *Polhillia Pallens*

Actinobacteria dominated in *P. pallens* rhizosphere soils in both dry (37.26%) and wet (39.3%) seasons, followed by unclassified bacteria with 16.8 and 19.1% in soils from the dry and wet seasons, respectively. Actinobacteria (37.2%), Firmicutes (8.9%), Verrucomicrobia (6.8%), Proteobacteria (5.4%) and Acidobacteria (5.0%) were the five most dominant phyla in soils collected during the dry season. In contrast, Chloroflexi (10.8%), Bacteroidetes (9.0%), Proteobacteria (6.1%), Planctomycetes (3.8%) and Firmicutes (3.4%) dominated in soils from the wet season. Apart from the variation in density, the bacterial populations were similar for the two seasons, except that Ascomycota was present in only the dry season and Cyanobacteria in only the wet season (Figure 2).

At the genus level, unclassified bacteria were 21.8% in the dry season and 20.2% in the wet season. *Conexibacter*, *Mycobacterium*, *Nocardioides* and *Chthoniobacter,* however, showed a high number of sequences in both seasons. Together, they accounted for 19.1% and 24.3% of the bacterial communities in the dry and wet seasons, respectively. *Candidatus Solibacter* (3.7%), *Clostridium* (3.4%) and *Thermoleophilum* (1.9%) were only present in soil from the dry season, while *Cicer* (2.9%), *Pseudonorcadia* (1.7%) and *Kribbella* (1.7%) were only found in soil from the wet season (Figure 3). The rarefaction analysis showed that about 2500 species were present in *P. pallens* rhizosphere during both wet and dry seasons based on 160,000 sequence reads obtained (Figure S1).

#### 3.4.3. *Wiborgia Sericea*

As found with the two *Polhillia* species, Actinobacteria dominated in the rhizosphere of *W. sericea* and, respectively, formed 44.6 and 37.7% of the sequences in the wet and dry seasons. Unclassified bacteria were the second largest group in both seasons (18.7 and 19.0%). Although their population sizes differed significantly between seasons, the five major phyla found in *W. sericea* rhizosphere soil were Chloroflexi (8.1 and 5.4%), Bacteroidetes (6.9 and 10.0%), Firmicutes (4.7 and 7.4%) and Proteobacteria (4.6 and 8.6%) during the dry and wet seasons, respectively. However, all the phyla were present in soils collected from the two seasons, except for Synergistetes (0.22%) and Cyanobacteria (0.85%), which were only detected in soil from the dry and wet seasons, respectively (Figure 2).

About 26.6% of the bacteria belonged to unclassified genera. Of the classified group, *Mycobacterium* was dominant in both dry (10.6%) and wet (9.4%) seasons, followed by *Dehalogenimonas* and *Conexibacter* in both dry (6.6% and 4.0%) and wet (3.6% and 3.7%) seasons, respectively. *Isosphaera* (5.4%) was present in only soil from the dry season, and *Bacillus* (3.7%) in only the wet season. Of the 13 classified bacterial genera listed, six were found in both wet and dry seasons (Figure 3). Species analysis using the rarefaction tool revealed 2000 species out of 160,000 sequences studied (Figure S1).

#### 3.4.4. *Wiborgia Obcordata*

During the two seasons, Actinobacteria (33.34–39.34%) was most dominant, followed by an unclassified phylum (18.5–19.1%) and Chloroflexi (9.8–10.8%). In the dry season, 9.01% and 6.1% of the bacteria belonged to Bacteroidetes and Proteobacteria, respectively, while in the wet season, 7.9% and 6.4% were Proteobacteria and Firmicutes phyla, respectively. Cyanobacteria were present in only soil collected during the dry season and Basidiomycota in only the wet season (Figure 2).

As found with the other species, *W. obcordata* rhizosphere soil recorded a much higher number of unclassified bacterial genera in both seasons (27.7% and 24.2%). The genera *Dehalogenimonas* (6.0%), *Ktedonobacter* (5.6%) and *Isosphaera* (5.0%) were dominant in the dry season, while *Conexibacter* (6.7%), *Dehalogenimonas* (6.6%) and *Mycobacterium* (5.8%) were higher in the wet season (Figure 3). The rarefaction studies showed that, of a total number of 160,000 sequences, only 1600 bacterial species were found in the dry season and 2000 species in the wet season (Figure S1).

#### 3.4.5. *Wiborgiella Sessilifolia*

With *W. sessilifolia*, only the dry season soil sequence results were included due to the poor concentration of DNA from soils collected in the wet season. Over half (56.2%) of the bacteria from dry season soil of *W. sessilifolia* belonged to Actinobacteria. Unclassified bacteria formed 12.08% of the total sequences, followed by Firmicutes (6.8%), Bacteroidetes (6.6%) and Proteobacteria (4.4%) (Figure 2). At the genus level, *Mycobacterium* (15.3%), *Pseudonocardia* (5.4%) and *Conexibacter* (4.7%) were dominant in the dry season after the unclassified genera (16.9%) (Figure 3). The number of species detected using 120,000 sequences was 1800 for soils sampled in the dry season (Figure S1).

## 4. Discussion

The microbial community structure in the fynbos is rather complex and includes bacteria, fungi and archaea. These microbes play a vital role in supporting plant growth in the acidic, nutrient-poor soils of the fynbos [28,31]. This study was conducted to ascertain any changes in microbial community structure during wet and dry seasons, and the results showed that the phylum Actinobacteria was very dominant (≥30%) in the rhizosphere of *Polhillia*, *Wiborgia* and *Wiborgiella* species during both wet and dry seasons. More specifically, up to 56.2% of the bacteria belonged to this Actinobacteria phylum in the rhizosphere soil of *Wiborgiella sessilifolia*. The dominance of Actinobacteria in the rhizospheres of the five shrub legumes could suggest the presence of these bacteria in the entire fynbos ecosystem. Actinobacteria have been reported to occur in abundance in rhizosphere soils from other biomes [32,33]. In fact, the phylum Actinobacteria has been reported to account for a large proportion of the microbial population in the rhizosphere of many plant species [34] and is also known to colonize root nodules of legumes, including lupin [35] An earlier study found that the phylum Actinobacteria was more abundant in undisturbed ecosystems than farmlands [36], a finding consistent with results from this study, which was conducted on undisturbed sites in the fynbos. The Actinobacteria phylum plays a major role in plant growth promotion through processes such as biological N_2_ fixation, production of IAA, antibiotics and siderophores, as well as the solubilization of P, K and Zn [37,38,39]. In fact, antibiotic resistance, the production of IAA and the solubilization of P are common functional attributes of bacterial symbionts associated with *Polhillia*, *Wiborgia* and *Wiborgiella* species [13]. 

Although their dominance differed per species and season, Bacteroidetes, Acidobacteria, Proteobacteria and Firmicutes were the major bacterial phyla with a higher number of sequences in the rhizosphere soils of the five legumes tested, a finding consistent with the results of earlier studies in the Cape fynbos [23,40]. This suggests that the rhizosphere microbial communities of the *Polhillia*, *Wiborgia* and *Wiborgiella* species are very diverse and high in numbers. Similar high diversity of bacterial community has been found in the rhizosphere of crops growing in undisturbed forest soil when compared to cultivated fields [41]. 

This study also discovered Acidobacteria in the rhizospheres of *Polhillia brevicalyx*, *Polhillia pallens, Wiborgia sericea* and *Wiborgia obcordata*, and this was not surprising given the acidic nature of the test rhizospheres (pH 4.3 to 5.5). Other studies similarly found Acidobacteria in the acidic and low-nutrient soils of the Cape fynbos [25,42]. It was, however, intriguing that the rhizosphere soil of *Wiborgiella sessilifolia*, with an alkaline pH of 8.2, could harbor up to 2.0% of Acidobacteria. Other studies have, however, also found Acidobacteria in alkaline habitats, including water [43]. 

In this study, a high number of unclassified bacterial genera were detected in the rhizosphere soils studied, a finding constant with earlier reports that found an abundance of uncultured microbes in soils from various study sites [22]. Of the classified bacteria, *Mycobacterium* and *Conexibacter* genera were more abundant in the rhizospheres of all test legumes during both wet and dry seasons, except for rhizosphere soil of *Wiborgia obcordata* collected during the dry season. *Mycobacterium*, and more specifically *Mycobacterium flavum*, has been reported to improve the growth of many crop species, including maize, wheat, sunflower, chickpea, cotton, soybean and rice [44,45,46,47,48]. *Conexibacter* was also dominant in the rhizosphere soil of sunflowers in South Africa [49]. *Dehalogenimonas* from the phylum Chloroflexi dominated the rhizosphere of *W. obcordata* during both wet (6.6%) and dry (6.0%) seasons and could play a significant role in plant defense against pathogenic microbes in the soil [50,51]. The high populations of plant growth-promoting bacteria found in the rhizosphere of legumes endemic to the Cape fynbos could explain the ability of these legumes to thrive under the nutrient-poor conditions of the fynbos [8].

Furthermore, the presence of N_2_-fixing *Burkholderia* and *Dehalogenimonas* bacteria in the acidic rhizospheres of the two *Wiborgia* species (Table 2; Figure 2) but not in the acidic rhizospheres of the two *Polhillia* species (Table 2; Figure 1) during the dry season, and the absence of *Streptomyces* and *Candidatus koribacter* during the wet season, could suggest the effect of plant species in shaping the structure of rhizosphere microbial communities in the fynbos [52]. The *Wiborgia* species probably released antimicrobial compounds that select for a specific type of microbiome in the rhizosphere [22]. The presence of *Burkholderia* in the fynbos has been associated with acidic soils, and the bacterium is known to fix N_2_ when in symbiosis with compatible legume hosts [5]. The fact that *Bacillus* and *Bradyrhizobium* were found in the rhizosphere of only *P. brevicalyx*, but not the other test legume species, and *Burkholderia* detected in the rhizospheres of only *W. sericea* and *W. obcordata*, attest to the effect of host plant in selecting its symbiont partner [53]. Furthermore, the effect of host plant is further supported by the nutrient content availability in the rhizosphere soil (Table 3). For instance, the two *Wiborgia* species (*W. obcordata and W. sericea*) contained relatively lower contents Mg, K, Na and P compared to *Polhillia* and *Wiborgiella* species.

*W. obcordata* was found to influence its rhizosphere more than the other test species during the two seasons. For example, although *Nocardioides* was absent in the rhizosphere of *W. obcordata* during both wet and dry seasons, it was present in the rhizosphere of the other four legumes. Furthermore, in the dry season, only the rhizosphere soil of *W. obcordata* contained populations of the plant growth-promoting *Bacillus* and *Bradyrhizobium* genera. However, in the wet season, the same soil harbored four unique bacterial genera (namely, *Ktedonobacter*, *Bradyrhizobium*, *Candidatus Solibacter* and *Acidobacterium*). These results probably suggest that *Wiborgia obcordata* released phytoalexins that were selected for a specific type of microbiome in the rhizosphere, a further indication that the microbial community in the rhizosphere soils is plant-regulated in the Cape fynbos [23,54,55]. 

Using partial chromosomal and symbiotic gene sequences, Mpai et al. [13] found a marked diversity of *Bradyrhizobium* bacteria present in the rhizosphere soil of *Wiborgia obcordata* at Bushmans Kloof. In fact, *Bradyrhizobium* was present in soil collected during the wet season (2.6%) but absent in soil from the dry season. The fact that this genus was absent in the microbial community during the dry season could suggest that this group of bacteria is highly endophytic and reside mainly inside root nodules, and available in the rhizosphere soils in very small numbers (˂0.5%), especially during the unfavorable dry seasons. Similarly, *Mesorhizobium, Rhizobium*, *Agrobacterium* and *Pseudomonas* bacteria, which were isolated from root nodules of these five shrub legumes [8,13], were also absent in this study, which is further evidence of the endophytic nature of these root-nodule bacteria.

Furthermore, the fact that *Bradyrhizobium* species were present in the rhizosphere soil of *W. obcordata* only in the wet but not the dry season could explain the increased nodulation, greater symbiotic functioning and high phosphatase secretion observed in this legume during the wet season in the Cape fynbos [56,57,58]. In fact, season and soil moisture have been found to be the main drivers of microbial community structure in both agricultural and natural ecosystems [59,60,61].

## 5. Conclusions

To our knowledge, this is the first report on total soil bacterial community structure associated with *Polhillia pallens*, *Polhillia brevicalyx*, *Wiborgia sericea*, *Wiborgia obcordata* and *Wiborgiella sessilifolia*, which are endemic to the Cape fynbos. In this study, plant species and season were the main drivers of bacterial community structure in the Cape fynbos. The wet season was more dominant in shaping microbial diversity relative to the dry season. Furthermore, our results also showed that *Wiborgia obcordata* had a greater influence on microbial community structure than the other four shrub legumes. Moreover, Actinobacteria was found to be the most common and dominant microbial phylum in the rhizosphere of all five test legumes regardless of season, followed by Bacteroidetes, Acidobacteria, Proteobacteria and Firmicutes. *Dehalogenimonas* was the major inhabitant of *Wiborgia obcordata* rhizosphere during the wet season, while *Mycobacterium* and *Conexibacter* were the major inhabitants in the rhizosphere soils of *P. pallens*, *P. brevicalyx* and *W. sericea* during both wet and dry seasons, as well as *W. sessilifolia* in the dry season. This study also found that a high number of bacteria in the Cape fynbos are still unclassified. Bacterial classification studies are therefore recommended to resolve the microbial community structure of legume rhizospheres in the Cape fynbos.

## Figures and Tables

**Table 1:** Summary of plant species and their sample sites.

Species	Collecting Site	Geographic Co-Ordinates	District
*Polhillia brevicalyx*	Ghwarriekop farm	34°33′53″S 19°59′43″E	Overberg
*Polhillia pallens*	Witkoppies farm	34°33′53″S 19°59′43″E	Overberg
*Wiborgia obcordata*	Bushmans Kloof	32°07′14″S 19°06′28″E	Cederberg
*Wiborgia sericea*	Traveller’s Rest farm	32°04′15″S 19°04′32″E	Cederberg
*Wiborgiella sessilifolia*	Bredasdorp/Elim Pass	34°37′58″S 19°49′399″E	Overberg

**Table 2:** Information on rhizosphere soil type, elevation and dry weight of soils obtained during the wet and dry seasons from *Polhillia*, *Wiborgia* and *Wiborgiella* species in the Cape fynbos biome of South Africa.

			Dry Season	Wet Season
Plant Species	Soil Type	Elevation (m)	*θ*_m_ Water Content (%)	
*Polhillia brevicalyx*	Clay	310	8.21 ± 0.00	25.47 ± 0.31
*Polhillia pallens*	Clay	152	9.26 ± 0.01	26.55 ± 0.06
*Wiborgia sericea*	Sandy	120	6.37 ± 0.01	20.00 ± 0.00
*Wiborgia obcordata*	Sandy	441	3.93 ± 0.05	11.90 ± 0.03
*Wiborgiella sessilifolia*	Loam	311	5.42 ± 0.03	

**Table 3:** Wet season rhizosphere soil chemical properties of *Polhillia, Wiborgia* and *Wiborgiella species* sampled from five different locations of the South African Cape fynbos in 2017.

pH and Minerals (mg/kg)	*Polhillia brevicalyx*	*Polhillia* *pallens*	*Wiborgiella sessilifolia*	*Wiborgia obcordata*	*Wiborgia* *sericea*
	**Concentrations**				
pH(H_2_O)	5.53 ± 0.03	4.45 ± 0.05	8.20 ± 0.00	4.33 ± 0.09	4.47 ± 0.09
Magnesium	4.55 ± 0.02	2.67 ± 0.01	3.08 ± 0.01	0.25 ± 0.01	0.27 ± 0.01
Potassium	170.18 ± 4.83	234.00 ± 1.00	53.67 ± 1.76	27.33 ± 1.76	37.00 ± 0.58
Sodium	110.22 ± 3.42	130.00 ± 0.00	64.33 ± 0.88	4.33 ± 0.33	9.00 ± 0.00
Phosphorus	22.00 ± 0.00	43.50 ± 0.50	15.67 ± 0.33	6.00 ± 0.58	8.33 ± 0.33
Copper	0.84 ± 0.00	0.46 ± 0.02	0.12 ± 0.01	0,14 ± 0.00	0.14 ± 0.1
Zinc	7.28 ± 0.05	2.84 ± 0.17	0.41 ± 0.01	0.18 ± 0.01	0.66 ± 0.03
Boron	10.67 ± 8.92	0.61 ± 0.01	0.80 ± 0.05	0.07 ± 0.00	0.12 ± 0.1
Carbon (%)	4.22 ± 0.13	7.76 ± 0.04	2.82 ± 0.15	0.39 ± 0.02	0.38 ± 0.02
Iron	105.85 ± 0.45	174.60 ± 6.20	46.98 ± 0.05	66.19 ± 2.88	129.2 ± 5.45
NH_4_^+^ (%)	0.40 ± 0.01	0.60 ± 0.00	0.67 ± 0.48	0.04 ± 0.01	0.03 ± 0.00

## Supplementary Materials

The following are available online at https://www.mdpi.com/article/10.3390/microorganisms10101992/s1, Figure S1: Rarefaction analysis bacterial species associated with *Polhillia brevicalyx, Polhillia pallens, Wiborgia sericea, Wiborgia obcordata* and *Wiborgiella sessilifolia* rhizosphere soil; Table S1: Information about the number and quality of sequences generated.

## References

[1] Forest F., Colville J.F., Cowling R.M. (2018). Evolutionary diversity patterns in the Cape flora of South Africa. Phylogenetic Diversity.

[2] Cowling R.M., Pressey R.L., Rouget M., Lombard A.T. (2003). A conservation plan for a global biodiversity hotspot—The Cape Floristic Region, South Africa. Biol. Conserv..

[3] Forest F., Nänni I., Chase M.W., Crane P.R., Hawkins J.A. (2007). Diversification of a large genus in a continental biodiversity hotspot: Temporal and spatial origin of Muraltia (Polygalaceae) in the Cape of South Africa. Mol. Phylogenet. Evol..

[4] Boatwright J.S., Cupido C.N. (2011). *Aspalathus crewiana* sp. nov.(Crotalarieae, Fabaceae) from the Western Cape Province, South Africa. Nord. J. Bot..

[5] Lemaire B., Dlodlo O., Chimphango S., Stirton C., Schrire B., Boatwright S., Honnay O., Smets E., Sprent J., James E. (2015). Symbiotic diversity, specificity and distribution of rhizobia in native legumes of the Core Cape Subregion (South Africa). FEMS Microbiol. Ecol..

[6] Postma A., Slabbert E., Postma F., Jacobs K. (2016). Soil bacterial communities associated with natural and commercial *Cyclopia* spp.. FEMS Microbiol. Ecol..

[7] Brink C., Postma A., Jacobs K. (2017). Rhizobial diversity and function in rooibos (Aspalathus linearis) and honeybush (*Cyclopia* spp.) plants: A review. S. Afr. J. Bot..

[8] Mpai T., Jaiswal S.K., Dakora F.D. (2020). Accumulation of phosphorus and carbon and the dependency on biological N-2 fixation for nitrogen nutrition in Polhillia, Wiborgia and Wiborgiella species growing in natural stands in cape fynbos, South Africa. Symbiosis.

[9] Boatwright J.S., Tilney P.M., Van Wyk B.-E. (2010). Taxonomy of Wiborgiella (Crotalarieae, Fabaceae), a genus endemic to the greater Cape Region of South Africa. Syst. Bot..

[10] Ahmad M.H., Uddin M.R., McLaughlin W. (1984). Characterization of indigenous rhizobia from wild legumes. FEMS Microbiol. Lett..

[11] Howieson J.G., De Meyer S.E., Vivas-Marfisi A., Ratnayake S., Ardley J.K., Yates R.J. (2013). Novel Burkholderia bacteria isolated from Lebeckia ambigua–a perennial suffrutescent legume of the fynbos. Soil Biol. Biochem..

[12] Lemaire B., Van Cauwenberghe J., Verstraete B., Chimphango S., Stirton C., Honnay O., Smets E., Sprent J., James E.K., Muasya A.M. (2016). Characterization of the papilionoid–Burkholderia interaction in the Fynbos biome: The diversity and distribution of beta-rhizobia nodulating Podalyria calyptrata (Fabaceae, Podalyrieae). Syst. Appl. Microbiol..

[13] Mpai T., Jaiswal S.K., Cupido C.N., Dakora F.D. (2021). Ecological adaptation and phylogenetic analysis of microsymbionts nodulating Polhillia, Wiborgia and Wiborgiella species in the Cape fynbos, South Africa. Sci. Rep..

[14] Cowling R.M., Procheş Ş., Partridge T.C. (2009). Explaining the uniqueness of the Cape flora: Incorporating geomorphic evolution as a factor for explaining its diversification. Mol. Phylogenet. Evol..

[15] Muofhe M.L., Dakora F.D. (1999). Nitrogen nutrition in nodulated field plants of the shrub tea legume Aspalathus linearis assessed using 15 N natural abundance. Plant Soil.

[16] Cramer M.D. (2020). Phosphate as a Limiting Resource: Introduction. Plant Soil.

[17] Maseko S.T., Dakora F.D. (2013). Rhizosphere acid and alkaline phosphatase activity as a marker of P nutrition in nodulated Cyclopia and Aspalathus species in the Cape fynbos of South Africa. S. Afr. J. Bot..

[18] Kamutando C.N., Vikram S., Kamgan-Nkuekam G., Makhalanyane T.P., Greve M., Le Roux J.J., Richardson D.M., Cowan D., Valverde A. (2017). Soil nutritional status and biogeography influence rhizosphere microbial communities associated with the invasive tree Acacia dealbata. Sci. Rep..

[19] Moroenyane I., Chimphango S.B.M., Wang J., Kim H.K., Adams J.M. (2016). Deterministic assembly processes govern bacterial community structure in the Fynbos, South Africa. Microb. Ecol..

[20] Wahdan S.F.M., Heintz-Buschart A., Sansupa C., Tanunchai B., Wu Y.-T., Schädler M., Noll M., Purahong W., Buscot F. (2021). Targeting the active rhizosphere microbiome of trifolium pratense in grassland evidences a stronger-than-expected belowground biodiversity-ecosystem functioning link. Front. Microbiol..

[21] Alkorta I., Epelde L., Garbisu C. (2017). Environmental parameters altered by climate change affect the activity of soil microorganisms involved in bioremediation. FEMS Microbiol. Lett..

[22] Jaiswal S.K., Mohammed M., Dakora F.D. (2019). Microbial community structure in the rhizosphere of the orphan legume Kersting’s groundnut [Macrotyloma geocarpum (Harms) Marechal & Baudet]. Mol. Biol. Rep..

[23] Zhou Y., Zhu H., Fu S., Yao Q. (2017). Variation in soil microbial community structure associated with different legume species is greater than that associated with different grass species. Front. Microbiol..

[24] Jaiswal S.K., Naamala J., Dakora F.D. (2018). Nature and mechanisms of aluminium toxicity, tolerance and amelioration in symbiotic legumes and rhizobia. Biol. Fertil. Soils.

[25] Brink C.J., Postma A., Slabbert E., Postma F., Muasya A.M., Jacobs K. (2020). Bacterial communities associated with natural and commercially grown rooibos (Aspalathus linearis). Pedosphere.

[26] Slabbert E., Kongor R.Y., Esler K.J., Jacobs K. (2010). Microbial diversity and community structure in Fynbos soil. Mol. Ecol..

[27] Moroenyane I., Chimphango S.B.M., Dong K., Tripathi B., Singh D., Adams J.M. (2019). Neutral models predict biogeographical patterns of soil microbes at a local scale in Mediterranean heathlands, South Africa. Trans. R. Soc. S. Afr..

[28] Jacobs K., Conradie T., Jacobs S. (2020). Microbial communities in the fynbos region of South Africa: What happens during woody alien plant invasions. Diversity.

[29] Zhang F., Zhou G. (2019). Estimation of vegetation water content using hyperspectral vegetation indices: A comparison of crop water indicators in response to water stress treatments for summer maize. BMC Ecol..

[30] Klindworth A., Pruesse E., Schweer T., Peplies J., Quast C., Horn M., Glöckner F.O. (2013). Evaluation of general 16S ribosomal RNA gene PCR primers for classical and next-generation sequencing-based diversity studies. Nucleic Acids Res..

[31] Mabiala S.T., Joseph G., Lebonguy A.A., Banga A.B. (2020). Diversity of the Bacterial Community of Three Soils Revealed by Illumina-Miseq Sequencing of 16S rRNA Gene in the South of Brazzaville, Congo. Am. J. Microbiol. Res..

[32] Nessner Kavamura V., Taketani R.G., Lançoni M.D., Andreote F.D., Mendes R., de Melo I. (2013). Water regime influences bulk soil and rhizosphere of Cereus jamacaru bacterial communities in the Brazilian Caatinga biome. PLoS ONE.

[33] Aguirre-Garrido J.F., Montiel-Lugo D., Hernández-Rodríguez C., Torres-Cortes G., Millán V., Toro N., Martínez-Abarca F., Ramírez-Saad H.C. (2012). Bacterial community structure in the rhizosphere of three cactus species from semi-arid highlands in central Mexico. Antonie Van Leeuwenhoek.

[34] Yadav A.N., Verma P., Kumar S., Kumar V., Kumar M., Sugitha T.C.K., Singh B.P., Saxena A.K., Dhaliwal H.S. (2018). Actinobacteria from rhizosphere: Molecular diversity, distributions, and potential biotechnological applications. New and Future Developments in Microbial Biotechnology and Bioengineering.

[35] Trujillo M.E., Riesco R., Benito P., Carro L. (2015). Endophytic actinobacteria and the interaction of Micromonospora and nitrogen fixing plants. Front. Microbiol..

[36] Acosta-Martinez V., Dowd S., Sun Y., Allen V. (2008). Tag-encoded pyrosequencing analysis of bacterial diversity in a single soil type as affected by management and land use. Soil Biol. Biochem..

[37] Le X.H., Franco C.M.M., Ballard R.A., Drew E.A. (2016). Isolation and characterisation of endophytic actinobacteria and their effect on the early growth and nodulation of lucerne (*Medicago sativa* L.). Plant Soil.

[38] Hardoim P.R., Andreote F.D., Reinhold-Hurek B., Sessitsch A., van Overbeek L.S., van Elsas J.D. (2011). Rice root-associated bacteria: Insights into community structures across 10 cultivars. FEMS Microbiol. Ecol..

[39] Chin K.-J., Hahn D., Hengstmann U.L.F., Liesack W., Janssen P.H. (1999). Characterization and identification of numerically abundant culturable bacteria from the anoxic bulk soil of rice paddy microcosms. Appl. Environ. Microbiol..

[40] Sharma P., Thakur D. (2020). Antimicrobial biosynthetic potential and diversity of culturable soil actinobacteria from forest ecosystems of Northeast India. Sci. Rep..

[41] Pereira R.M., da Silveira É.L., Scaquitto D.C., Pedrinho E.A.N., Val-Moraes S.P., Wickert E., Carareto-Alves L.M., Lemos E.G.d.M. (2006). Molecular characterization of bacterial populations of different soils. Braz. J. Microbiol..

[42] Conradie T., Jacobs K. (2020). Seasonal and agricultural response of Acidobacteria present in two fynbos rhizosphere soils. Diversity.

[43] Vieira S., Luckner M., Wanner G., Overmann J. (2017). Luteitalea pratensis gen. nov., sp. nov. a new member of subdivision 6 Acidobacteria isolated from temperate grassland soil. Int. J. Syst. Evol. Microbiol..

[44] Tarafdar J.C., Rathore I., Shiva V. (2012). Effect of Bt-transgenic cotton on soil biological health. Appl. Biol. Res..

[45] Oliveira C.A., Alves V.M.C., Marriel I.E., Gomes E.A., Scotti M.R., Carneiro N.P., Guimaraes C.T., Schaffert R.E., Sá N.M.H. (2009). Phosphate solubilizing microorganisms isolated from rhizosphere of maize cultivated in an oxisol of the Brazilian Cerrado Biome. Soil Biol. Biochem..

[46] Ambrosini A., Beneduzi A., Stefanski T., Pinheiro F.G., Vargas L.K., Passaglia L.M.P. (2012). Screening of plant growth promoting rhizobacteria isolated from sunflower (*Helianthus annuus* L.). Plant Soil.

[47] Verma P., Yadav A.N., Kazy S.K., Saxena A.K., Suman A. (2013). Elucidating the diversity and plant growth promoting attributes of wheat (Triticum aestivum) associated acidotolerant bacteria from southern hills zone of India. Natl. J. Life Sci..

[48] Verma P., Yadav A.N., Kazy S.K., Saxena A.K., Suman A. (2014). Evaluating the diversity and phylogeny of plant growth promoting bacteria associated with wheat (Triticum aestivum) growing in central zone of India. Int. J. Curr. Microbiol. Appl. Sci..

[49] Babalola O.O., Alawiye T.T., Rodriguez Lopez C.M., Ayangbenro A.S. (2020). Shotgun metagenomic sequencing data of sunflower rhizosphere microbial community in South Africa. Data Brief.

[50] Maphosa F., de Vos W.M., Smidt H. (2010). Exploiting the ecogenomics toolbox for environmental diagnostics of organohalide-respiring bacteria. Trends Biotechnol..

[51] Pan Y., Chen J., Zhou H., Farzana S., Tam N.F.Y. (2017). Vertical distribution of dehalogenating bacteria in mangrove sediment and their potential to remove polybrominated diphenyl ether contamination. Mar. Pollut. Bull..

[52] Schmidt J.E., Kent A.D., Brisson V.L., Gaudin A.C.M. (2019). Agricultural management and plant selection interactively affect rhizosphere microbial community structure and nitrogen cycling. Microbiome.

[53] Puozaa D.K., Jaiswal S.K., Dakora F.D. (2019). Phylogeny and distribution of *Bradyrhizobium* symbionts nodulating cowpea (*Vigna unguiculata* L. Walp) and their association with the physicochemical properties of acidic African soils. Syst. Appl. Microbiol..

[54] Miyambo T., Makhalanyane T.P., Cowan D.A., Valverde A. (2016). Plants of the fynbos biome harbour host species-specific bacterial communities. FEMS Microbiol. Lett..

[55] Turner T.R., James E.K., Poole P.S. (2013). The plant microbiome. Genome Biol..

[56] Peoples M.B., Brockwell J., Hunt J.R., Swan A.D., Watson L., Hayes R.C., Li G.D., Hackney B., Nuttall J.G., Davies S.L. (2013). Factors affecting the potential contributions of N2 fixation by legumes in Australian pasture systems. Crop. Pasture Sci..

[57] Pimratch S., Jogloy S., Vorasoot N., Toomsan B., Patanothai A., Holbrook C.C. (2008). Relationship between Biomass Production and Nitrogen Fixation under Drought-Stress Conditions in Peanut Genotypes with Different Levels of Drought Resistance. J. Agron. Crop. Sci..

[58] Parvin S., Uddin S., Bourgault M., Roessner U., Tausz-Posch S., Armstrong R., O’Leary G., Fitzgerald G., Tausz M. (2018). Water availability moderates N2 fixation benefit from elevated [CO_2_]: A 2-year free-air CO_2_ enrichment study on lentil (*Lens culinaris* MEDIK.) in a water limited agroecosystem. Plant. Cell Environ..

[59] Taketani R.G., Lançoni M.D., Kavamura V.N., Durrer A., Andreote F.D., Melo I.S. (2017). Dry season constrains bacterial phylogenetic diversity in a semi-arid rhizosphere system. Microb. Ecol..

[60] Brockett B.F.T., Prescott C.E., Grayston S.J. (2012). Soil moisture is the major factor influencing microbial community structure and enzyme activities across seven biogeoclimatic zones in western Canada. Soil Biol. Biochem..

[61] Liu J., Chen X., Shu H., Lin X., Zhou Q., Bramryd T., Shu W., Huang L. (2018). Microbial community structure and function in sediments from e-waste contaminated rivers at Guiyu area of China. Environ. Pollut..

