# Supplementary material for: Seasonal Effect on Bacterial Communities Associated with the Rhizospheres of Polhillia, Wiborgia and Wiborgiella Species in the Cape Fynbos, South Africa
Source: Microorganisms. 2022 Oct 9;10(10):1992. doi: 10.3390/microorganisms10101992 (PMC9612010; doi:10.3390/microorganisms10101992)
Supplement: Supplementary file 1 [file microorganisms-10-01992-s001.zip › microorganisms-1889723-supplementary.pdf]

Species	Dry season	Wet season
Polhillia brevicalyx	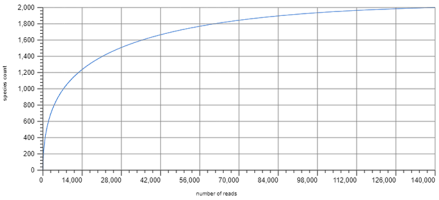	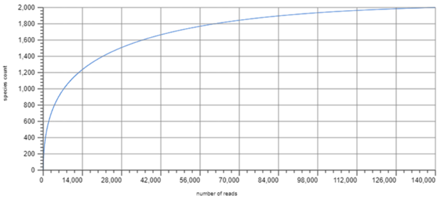
Polhillia pallens	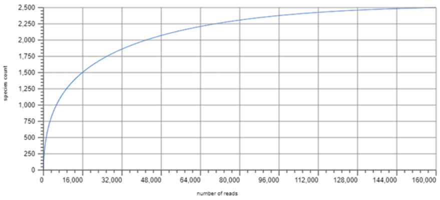	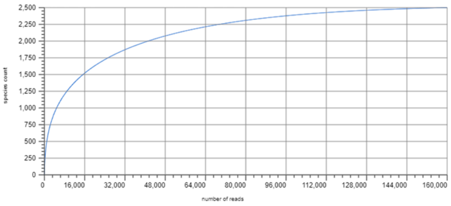
Wiborgia sericea	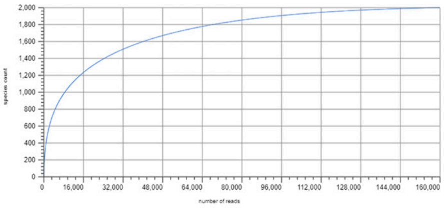	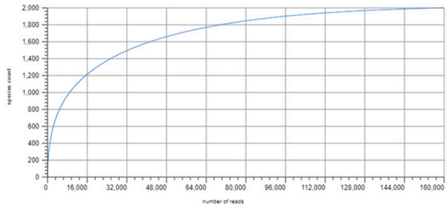
Wiborgia obcordata	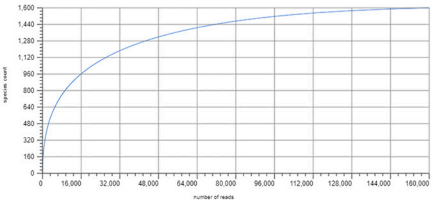	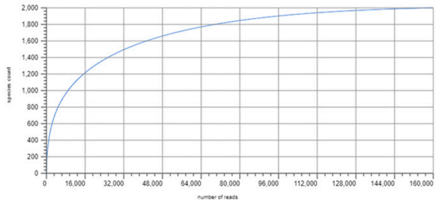
Wiborgiella sessilifolia	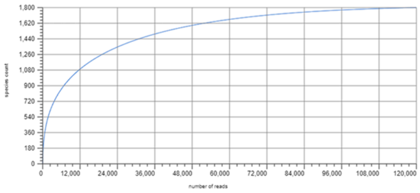	

Figure S1: Rarefaction analysis bacterial species associated with *Polhillia brevicalyx, Polhillia pallens, Wiborgia sericea, Wiborgia obcordata* and *Wiborgiella sessilifolia* rhizosphere soil.

Table S1: Information about the number and quality of sequences generated

Season	Plant species	Sample site	Total bases (bp)*	Raw reads (bp)*	GC (%)	Q20 (%)	Q30 (%)
**Dry**							
	Polhillia brevicalyx	Ghwarriekop farm	4444959	11820	57±4	93.13	85.84
	Polhillia pallens	Witkoppies	5537882	14904	58±5	92.47	84.84
	Wiborgia sericea	Traveller's Rest Farm	5180741	14305	58±5	91.97	84.01
	Wiborgia obcordata	Bushmans kloof	4882646	13485	58±5	91.9	83.92
	Wiborgiella sesilifollia	Bredasdorp	4521971	12006	57±3	92.1	84.03
**Wet**							
	Polhillia brevicalyx	Ghwarriekop farm	5072395	13536	57±4	92.69	85.15
	Polhillia pallens	Witkoppies	5792049	15764	58±5	92.53	84.94
	Wiborgia sericea	Traveller's Rest Farm	5459978	14819	58±5	92.77	83.31
	Wiborgia obcordata	Bushmans kloof	5771647	15164	58±5	92.71	85.15
